# F-18-FDG PET/CT findings of paraneoplastic dermatoses

**DOI:** 10.1007/s11604-022-01286-x

**Published:** 2022-06-17

**Authors:** Kazuyoshi Suga

**Affiliations:** Department of Radiology, St. Hill Hospital, 3-7-18 Imamurakita, Ube, Yamaguchi 755-8505 Japan

**Keywords:** Paraneoplastic dermatoses, F-18-FDG PET/CT, Malignant tumors

## Abstract

Paraneoplastic dermatoses (PD) are defined as nonspecific skin disorders which are associated with internal neoplasms, but without direct association to primary tumors or metastases. Recognition of PD and the following surveillance may lead to the diagnosis of internal malignant neoplasms including early stage ones. Accurate imaging examinations in the following searching is essential in identifying the underlying neoplasms. Since whole-body 18-fluoro-2-deoxyglucose (F-18-FDG)-positron emission (PET)/computed tomography (CT) has been widely used in early diagnosis, staging of various malignant tumors, it may play a role for detection of underlying or occult malignant neoplasms in patients with PD. However, to date, only a few reports of FDG PET/CT findings of the associated neoplasms in PD patients have been cited in the literature. The present paper shows the cases of FDG-avid associated neoplasms in patients with PD in our 10-year experience in our institute, and reviews the well-known and/or relatively common PD and their associated neoplasms, and the previously reported cases of FDG-avid associated neoplasms in these patients.

## Introduction

Paraneoplastic syndromes are defined as clinical syndromes involving non-metastatic systemic effects that accompany malignant diseases [1–3]. These syndromes are collections of symptoms that result from substances produced or induced by the tumor, and they occur remotely from the tumor itself [1]. They are consisted of heterogeneous disorders that can affect any organ system including the central and peripheral nervous systems as well as the musculoskeletal, dermatologic, hematologic, endocrine, or gastrointestinal systems [1–3]. Among these disorders, paraneoplastic dermatoses (PD) are generally defined as nonspecific skin disorders which are associated with internal neoplasms [4–9]. PD are relatively rare, acquired diseases, and to date, over 50 PD have been reported [8]. PD may be caused by a variety of factors related to the internal neoplasms, such as polypeptides, hormones, cytokines, antibodies or growth factors that act as mediators, interfering with cell communication [4–9]. As well as other paraneoplastic syndromes, PD can be potential markers or a warning sign of internal or occult malignancy. Recognition of PD and the following surveillance may lead to the early diagnosis of internal malignancy and a better prognosis, since PD occasionally precede underlying neoplasms. Whole-body 18-fluoro-2-deoxyglucose (F-18-FDG)-positron emission (PET)/computed tomography (CT) can detect the various malignant tumors with increased cellular metabolism by showing high FDG uptake even in patients with cancer of unknown primary origin, and may also contribute to the detection of underlying neoplasms in patients with PD. Although the previous investigators have shown some promising results in the role of whole-body FDG PET/CT in the workup of patients with suspected internal neoplasms and paraneoplastic syndromes [1–3], to date, only a few reports of FDG-avid-associated neoplasms in PD patients have been cited in the literature. This paper shows the cases of FDG-avid-associated neoplasms in PD patients which were experienced during the past 10 years in our institute, and also reviews the well-known and/or relatively common PD and their associated neoplasms based on the literature, and the previously reported cases of FDG-avid-associated neoplasms in PD patients.

## Classification of PD

Based on pathological aspect, PD can be classified in papulosquamous disorders, interface dermatitis, reactive erythema, neutrophilic dermatosis, dermal proliferating disorder, deposition disorder and others (Table 1) [4–9]. Based on the frequency in which PD is associated with neoplasms, PD are classified in two groups: obligate PD, in which the associated neoplasm is present in 90–100% of the cases, and facultative PD, in which the neoplasm can be detected in 25–30% of the cases [5]. There are also some other skin diseases which the clinicians frequently encounter and could be associated with underlying neoplasms, but with less associative strength compared with other PD [5, 7, 9]. In this paper, we describe PD according to the frequency in which PD is associated with underlying neoplasms, and also briefly describe other common skin diseases which could be classified as PD in a broad sense.

**Table 1:** Classification of PD based on pathological aspect

Paraneoplastic dermatoses	Associated neoplasms
Papulosquamous disorder	
Acanthosis nigricans	Gastric, esophageal, pancreatic, liver and bile duct adenocarcinomas
Acquired pachydermatoglyphia	Gastric and pulmonary carcinomas, esophageal carcinomas
Leser–Trélat sign	Gastric and colorectal adenocarcinomas, esophagus, duodenum, pancreas, gallbladder and liver carcinomas
Bazex syndrome	Aerodigestive tract carcinomas (oral cavity, larynx, pharynx, trachea, esophagus and lung)
Interface dermatitis	
Paraneoplastic pemphigus	Non-Hodgkin lymphoma, chronic lymphocytic leukemia, Castleman’s disease, and thymoma
Paraneoplastic dermatomyositis	Lower respiratory, gastrointestinal tract and ovarian carcinomas
Reactive erythema	
Necrolytic migratory erythema	Glucagonoma
Erythema gyratum repens	Pulmonary, esophageal and breast carcinomas
Neutrophilic dermatosis	
Pyoderma gangrenosum	Myelodysplastic syndrome, myeloma, and leukemia
Sweet syndrome	Myeloproliferative and lymphoproliferative disorders, and colorectal carcinomas
Dermal proliferating disorder	
Multicentricretic hystiocytosis	Gastric, ovarian, breast and uterine carcinomas, myeloma, melanoma and lymphoma
Necrobiotic xanthgranuloma	Multiple myeloma and chronic myelomonocytic leukemia
Deposition disorder	
Scleromyxedema	Multiple myeloma, Waldenström macroglobulinemia, Hodgkin and non-Hodgkin lymphomas, leukemia and thymic carcinoma
Cutaneous amyloidosis	Multiple myelomas
Others	
Acquired hypertricosis lanuginose	Colorectal, pulmonary and breast carcinomas

### Obligate PD

#### Acanthosis nigricans

Acanthosis nigricans (AN) typically develops suddenly in people over 40 years of age as symmetrical hyper-pigmentation in intertriginous areas, without differences by sex [5, 9–14]. AN is characterized by extensive condition, progressive course, pruritic, hyper-keratotic, and hyper-pigmented plaques with a subsequent formation of velvety papillomas, and the lesions are frequently involved in the skin wrinkles of the neck, armpits, groin, and axillary region [14]. AN often precedes or occurs simultaneously with the diagnosis of cancer. AN is most frequently associated with intra-abdominal carcinomas (73.2%), including gastric adenocarcinoma and other tumors in the liver, uterus/cervix, breast, lung, pancreas, and colon/rectum [5, 11]. In Japan, the frequency of gastric cancer is higher (more than 90%) compared with western countries [10]. Currently, the pathogenesis is proposed that certain cytokines produced by the tumor, such as transforming growth factor alpha, insulin-like growth factor 1, and fibroblast growth factor, participate in the development of the lesions in AN through the stimulation of keratinocytes, melanocytes, and fibroblasts [5, 14].

There is a case report of FDG-avid esophageal cancer detected by FDG PET/CT in a patient with AN [10]. The reported patient underwent FDG PET/CT subsequently after the diagnosis of AN, which showed an intense FDG uptake in the esophagus without any FDG-avid lymph nodes. The surgery revealed esophageal squamous cell carcinoma at the pathological stage of T3, N0 and M0. It is noteworthy that the dermal thickening and the pigmentation of AN were completely relieved at 6 months after the surgery in this patient.

#### Acquired pachydermatoglyphia, Acquired palm keratosis//Tripe palms, Leser-Trélat Sign

Acquired pachydermatoglyphia (AP) or acquired palm keratosis/tripe palms is dermatose that presents as hyperkeratosis of the palms and soles, with male predominance [8, 15–18]. AP is characterized by yellowish, velvety, diffuse palmar hyperkeratosis, with accentuated dermatoglyphic patterns, that resembles the intestinal mucosa. Pathologically, acanthosis, hyperkeratosis, and perivascular deposition of mucin in the dermis is shown. AP is usually associated with Leser–Trélat sign (LTS) described later and AN. Neoplastic processes have been reported in 90% of cases of AP, including gastric and lung cancers (50%) and breast and genitourinary tract cancers [8]. Physiologically, a pivotal role of epidermal growth factor-α and transforming growth factor-α released by neoplastic cells has been reported.

LTS shows a rapid appearance of multiple seborrheic keratoses on the thorax and dorsum, and occurs equally in men and women, and at an average age of 61 years [8, 15]. Approximately half of all cancers associated with LTS are adenocarcinomas, present in the gastrointestinal tract in 32% of cases [8]. Lymphoproliferative abnormalities are associated in 21% of cases. Because keratoses are benign and a common occurrence in older people, it is often ignored. It has been recommended that all patients with LTS should be screened for neoplasms [8, 15].

There is a case report of FDG-avid mediastinal squamous cell carcinoma detected by FDG PET/CT in a patient with LTS [15]. This patient had a hypermetabolic lesion in the region of the aortic arch on the mediastinal mass on FDG PET/CT, which was later pathologically diagnosed as a primary keratinizing squamous cell carcinoma of the lung.

#### Acrokeratosis paraneoplastica of Bazex (Bazex syndrome)

Bazex syndrome is characterized by psoriasiform changes on the digits, and in some patients spread to the ears, nose and in later stages to the limbs and trunk [8, 16–18]. This paraneoplastic process predominates in men with an average age of 40 years. As the disease progresses, desquamation affects the dorsal and palmoplantar regions producing a violaceous keratorderma. All the cases cited in the literature were associated with malignancy. Skin manifestations often precede the diagnosis of cancer in approximately 2–6 months in 65–70% of patients. About 80% of cases are associated with a squamous cell carcinoma of the upper aero-digestive tract [15, 16]. As the pathogenesis, the crossed reactions between tumor antigens and growth factor receptors on epidermal cells and/or the cutaneous changes by the action of epidermal growth factor, transforming growth factor alpha and insulin-like growth factor secreted by the tumor cells are proposed [15, 16].

There are case reports of FDG-avid nodular sclerosing Hodgkin disease and lung cancer in patients with Bazex syndrome [17, 18]. One of these patients showed diarrhea and weight loss, a colonoscopy was initially performed but it was normal. Subsequent FDG PET/CT revealed multiple FDG-avid retroperitoneal lymphadenopathies, which were diagnosed as nodular sclerosing Hodgkin disease by a punction of these lymphadenopathies. It is noteworthy that after two rounds of chemotherapy, the skin symptoms of Bazex syndrome had disappeared in this patient. In the other patient, FDG PET/CT showed FDG uptake in the lung tumor and mediastinal lymphadenopathy. A transbronchial biopsy of the lung tumor showed adenocarcinoma, which was diagnosed as stage IVB lung cancer.

#### Paraneoplastic pemphigus

Paraneoplastic pemphigus (PNP) is a clinically, histologically and immunologically distinct autoimmune muco-cutaneous disease, and is a rare skin condition involving severe blistering of the mucus membranes, most often affects people aged 45–70 years, without gender predominance [8, 19, 20]. PNP typically presents with painful mucosal erosions and dusky patches on the skin that later desquamate. The mouth is always affected, but other regions including the lips, oropharynx, nasopharynx, conjunctivae, anogenital region, and esophagus may also be affected, after which cutaneous lesions may appear. Auto-reactive T cells and IgG autoantibodies against heterogeneous antigens, including plakin family proteins and desmosomal cadherins, contribute to the pathogenesis of PNP. Two-thirds of patients with PNP have a recognized neoplasm at the onset of PNP. Approximately 80% of the associated neoplasms are of hematological origin, such as non-Hodgkin lymphoma (42%), chronic lymphocytic leukemia (29%), Castleman disease (10%), thymoma, Waldernstrom's macroglobulinemia and follicular dendritic cell sarcoma [8].

**Fig. 1:**
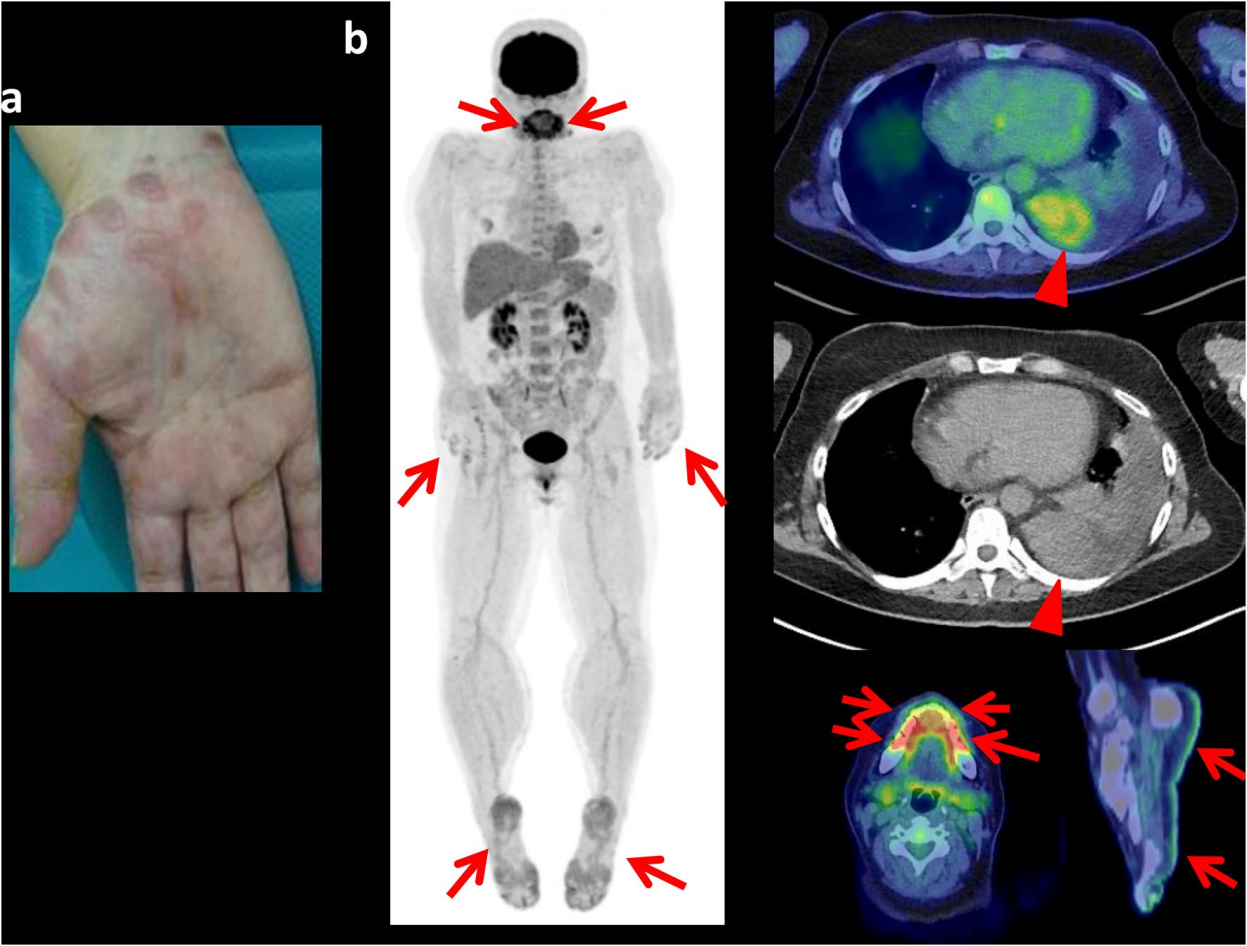
Cutaneous manifestation of paraneoplastic pemphigus at the hand in a 45 year-old female who presented with oral mucosal and lip lesions and polymorphous cutaneous eruption including the both hands and feet (**a**). Whole-body FDG PET maximum projection image showed abnormal FDG uptake (SUVmax 7.3) in the mouth/lips lesions, also abnormal uptakes (SUVmax6.9) in the both hands and feet (**b**; arrows). Cross-sectional FDG PET/CT images showed FDG-avid (SUVmax 4.0) mass in the left thorax (arrowhead) and abnormal FDG uptakes in the lip/mouth lesions and cutaneous lesion of the feet (arrows). Later, this patient had surgical resection of the thoracic FDG-avid mass which was diagnosed as hyaline vascular-type Castleman disease

There are several case reports of FDG-avid neoplasms (Castleman disease, mediastinal follicular dendritic cell sarcoma, and inflammatory myofibroblastic tumor) in patients with PNP [21–23]. In the case of the mediastinal sarcoma, FDG PET/CT showed intense F-FDG uptake in the primary tumor, accompanied by parasternal FDG-avid adenopathy. The surgical pathology proved parasternal lymph node metastasis. Mediastinal follicular dendritic cell sarcoma is very rare disease, and there are only 5 reports on the PET-CT features of mediastinal follicular dendritic cell sarcoma including the reported case, whereas SUVmax ranged from 2.7 to 11.4. While the reported case of inflammatory myofibroblastic tumor showed an intense FDG uptake, FDG uptake in this tumor generally vary from low to high FDG uptake, which may be due to tumor cellularity, biological behaviors of the tumor cells, the composition and the proportion of inflammatory cells, and the extent of activation of the inflammatory cells [23]. Our case showed FDG-avid thoracic Castleman disease, also with FDG-avid cutaneous PNP lesions (Fig. 1). FDG-avid cutaneous PNP lesion has not yet been described in the previous literature. As the presence of inflammatory cells in the cutaneous PNP lesion has been reported as one of the major histological features [8, 19, 20], FDG uptake by these inflammatory cells may cause FDG avidity in the cutaneous lesions.

#### Necrolytic migratory erythema

In necrolytic migratory erythema (NME), initially, a pinkish, maculopapular rash with irregular edges and a standard arcuate or polycyclic pattern, prominent in areas of trauma, is observed, often affecting the knees and intertriginous areas [24]. NME can represent an early sign of pancreatic glucagonoma [24]. The pathogenesis of NME has been reported that the reduced level of zinc and amino acids caused by tumor metabolism could determine an increase in arachidonic acid production, leading to cutaneous inflammation.

There is a case report of FDG-avid pancreatic glucagonoma on FDG PET/CT in a patient with NME [25]. In the reported case, mild FDG uptake with SUVmax of 2.3 was seen in the tumor.

#### Erythema gyratum repens

In erythema gyratum repens (EGR), the skin develops a widespread, serpiginous, polycyclic and pruriginous erythema which is desquamative around the edges, and fast-growing, producing concentric figures that resemble a wood surface that may cover much of the trunk and proximal extremities [24, 26]. ERG is seen more commonly in men, at an average age of 63 years, and malignant neoplasms are found in 82% of the patients with EGR. Lung cancer is the most common (32%), followed by cancer of the esophagus (8%) and breast (6%) [8]. Other malignancies also are associated with EGR, such as colon, stomach, bladder, prostate, uterine, rectal and pancreatic cancer and multiple myeloma [8]. The diagnosis of EGR precedes the diagnosis of the underlying neoplasms in approximately 80% of patients. As the pathogenesis of EGR, tumor antigens may form and cross-react with endogenous skin antigens or tumor products and may alter endogenous skin antigens making them susceptible to autoimmune recognition. EGR seems to be extremely rare, as a current literature search yielded a handful of additional case reports [24, 26].

There is a case report of FDG-avid T cell lymphoma on FDG PET/CT in a patient with EGR [27]. The reported patient presented with a solitary large mass with central necrosis on the occipital region, which showed abnormal FDG uptake.

#### Acquired hypertricosis lanuginose

Acquired hypertricosis lanuginose (AHL) is characterized by the sudden onset of thin and soft hair, lanugo-like, initially on the face predominantly in women [8, 28]. AHL is most commonly associated with adenocarcinomas of the lung and colon (27% and 24%, respectively) [8, 28]. AHL is extremely rare, and to date, a total of only 56 patients with AHL-associated neoplasms have been reported.

To our knowledge, there is no case report of FDG-avid neoplasms on FDG PET/CT in patients with AHL.

### Facultative PD

#### Pyoderma gangrenosum (PG)

Pyoderma gangrenosum (PG) is a rare, idiopathic, inflammatory, neutrophilic dermatosis that is generally characterized by recurrent sterile skin ulceration [29]. Up to 7% of PG cases are associated with underlying hematological neoplasms, such as myelodysplastic syndrome, myeloma, and acute myelogenousleukemia [8]. In addition, it can be associated with solid organ malignancy and rectal cancer. Pathologically, non-specific neutrophilic infiltration in the dermis is observed.

There is a case report of FDG-avid lymphoma on FDG PET/CT in a patient with PG [30]. In the reported patient, FDG PET/CT exhibited a nodular mass with intense FDG uptake in the right lower lung field, and extensive and marked FDG-avid sites in multiple sites of the bones and soft tissue of the left lower leg.

#### Sweet syndrome

Sweet syndrome (SS) is a prototypic acute febrile neutrophilic dermatosis, clinically characterized by painful, edematous, shiny erythematous nodules or plaques, which usually occur in the head, neck, and upper limbs. Pathologically, there are diffuse neutrophilic infiltrate in the dermis, edema, and fragmentation of the nuclei of neutrophils [8, 31–33]. Paraneoplastic SS accounts approximately for 21% of total SS cases; 85% of paraneoplastic SS are associated with hematological disorders, such as acute myelogenous leukemia, myelodysplastic syndrome, Hodgkin disease and polycythemia vera. In addition, paraneoplastic SS can be associated with adenocarcinomas of the breast, genitourinary tract and gastrointestinal tract. Although the paradigm of PD is the absence of the neoplastic cells into cutaneous lesion, an exception is represented by SS associated with a hemopoietic neoplasm, where myeloid cells are often detected in the cutaneous biopsy. In paraneoplastic SS, the over-production and dysregulation of inflammatory cytokines, like interleukin, granulocyte colony-stimulating factor, and granulocyte macrophage colony-stimulating factor have been shown to be involved in the development of SS [8].

**Fig. 2:**
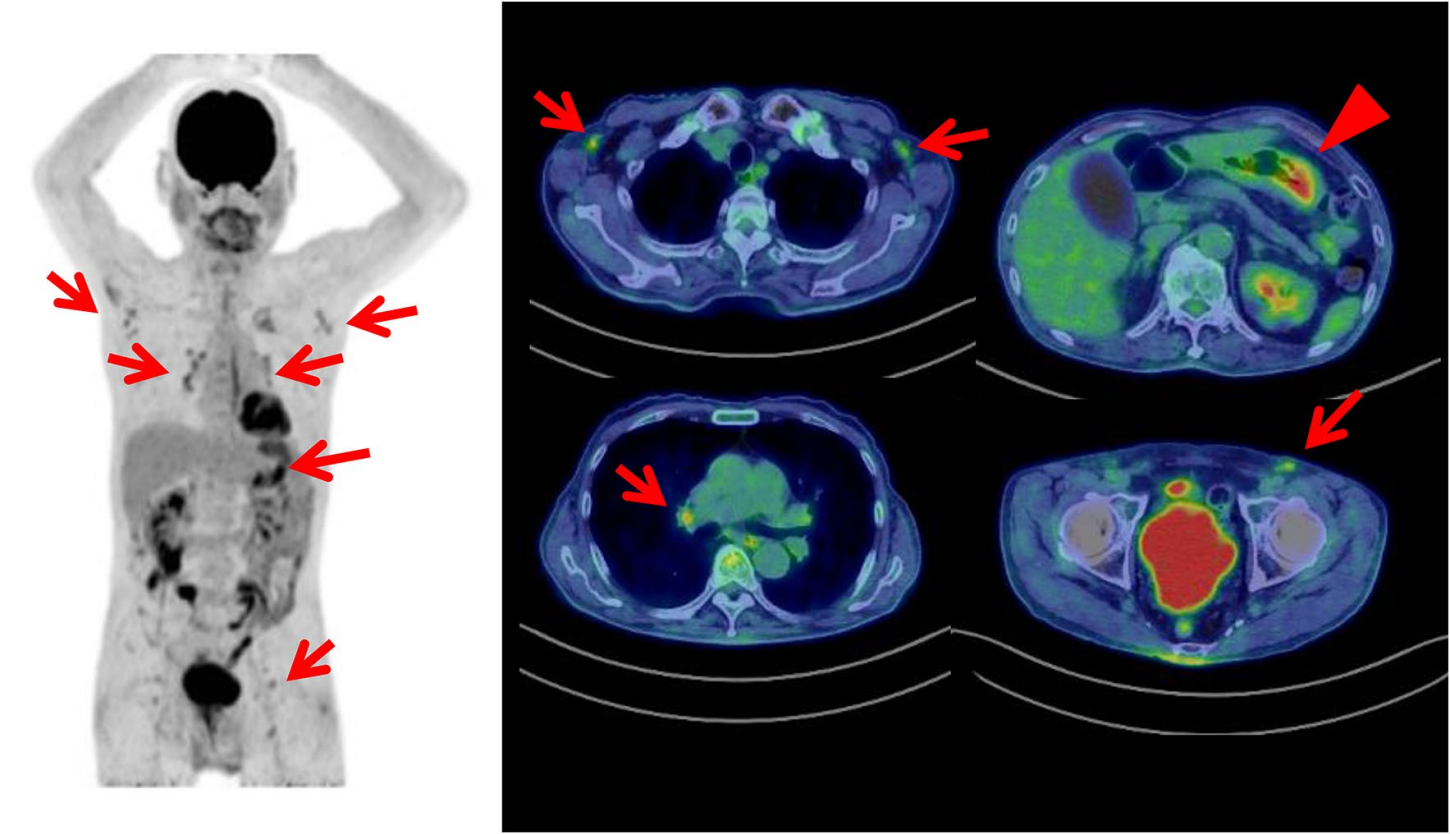
In a 76-year-old male with Sweet syndrome, whole-body FDG PET maximum projection and cross-sectional images showed FDG-avid (SUVmax 2.1) lymphadenopathy in the both axillary and hilar regions and in the both external iliac and left inguinal regions (arrows). The biopsy of the left inguinal lymph node revealed follicular lymphoma. In addition, focal intensive FDG uptake (SUVmax 5.0) was also seen in the lower part of gastric corpus (arrowhead), which was later diagnosed as gastric cancer by endoscopic biopsy

To date, the cases of FDG-avid myelodysplastic syndrome and non-Hodgkin T cell lymphoma on FDG PET/CT in patients with SS have been reported [31, 32]. In the reported patient of myelodysplastic syndrome, FDG PET/CT showed multiple cutaneous foci with increased FDG uptake throughout the body, which were some of the cutaneous erythematous nodules of SS [31]. FDG PET/CT also showed multiple FDG-avid lymph nodes in the mediastinum, bilateral pulmonary hili and abdomen, and diffusively increased FDG uptake of bone marrow. In the reported case of T cell lymphoma, FDG PET/CT showed hepatomegaly with intense FDG uptake, which was diagnosed as T cell lymphoma by a liver biopsy [32]. Our case of SS showed FDG-avid follicular lymphoma lesions and gastric cancer (Fig. 2).

#### Dermatomyositis

Dermatomyositis (DM) is an uncommon idiopathic, inflammatory myopathy characterized by proximal muscle weakness and cutaneous lesions. Skin lesions precede muscle involvement by months or years in more than 50% of DM patients　[8, 34–42]. As cutaneous lesions, a violet-colored or dusky red rash and a heliotrope rash develop, most commonly on face and eyelids and on knuckles, elbows, knees, chest and back. The rash can be itchy and painful. The reported rate of occurrence of malignant neoplasms in patients with DM is 15–30% [39]. Dysphagia occurs in 10–20% of patients. The risk of malignancy increases with the age of the patients, and is higher in the first year after diagnosis, then steadily decreases through five years, but remains persistently slightly elevated in comparison to the general population [8]. Predictive factors for malignant neoplasms in DM include patient age over 52 years, male gender, ulcers, skin necrosis, dysphagia, increased erythrocyte sedimentation rates, increased amounts of C-reactive protein, anti-155/140 or transcriptional intermediary factor 1 (TIF1) γ antibodies, and elevated serum creatine phosphokinase [8, 34–42]. While lung and gastrointestinal neoplasms are the mostly reported in DM patients, different malignancies including nasopharynx, ovarian, breast, prostate, kidney cancers, and different types of hematological malignancies are also reported. In Japan, gastric cancer is found in up to 25% of patients [37]. The etiopathogenesis of DM is still unclear, but recent studies have suggested that DM might arise as an autoimmune response against cancer which cross-reacts with regenerating muscle cells, as myositis-specific autoantigens are expressed in both tumor cells and undifferentiated myoblasts [34–42]. An association between autoantibody (anti-155/140 or transcriptional intermediary factor 1) IgG and paraneoplastic DM also has been described [35, 37].

Whole-body FDGPET/CT often shows symmetrical muscle hyper-metabolism representing the inflammatory nature of DM, with correlation with serum muscle enzymes in DM patients [37]. Proximal muscles of shoulders, buttocks and thigh are the most frequent FDG-positive regions.

**Fig. 3:**
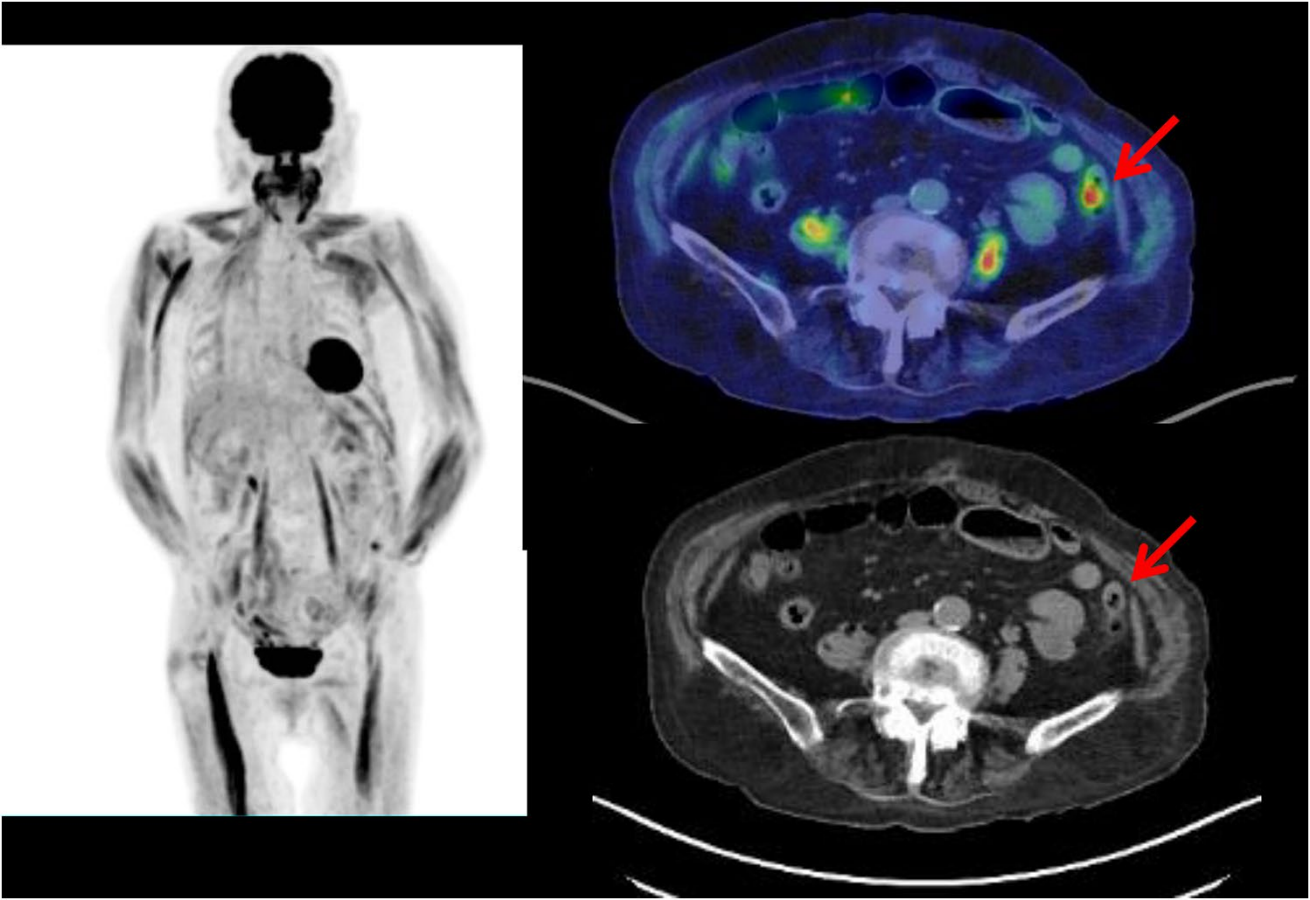
A 71-year-old female with transcriptional intermediary factor 1-positive dermatomyositis was treated with corticosteroid. This patient had a history of thyroidectomy for thyroid cancer 7 years ago. Whole-body FDG PET maximum projection image showed increased FDG uptake in systemic muscles. Cross-sectional FDG PET/CT images showed focal FDG uptake (SUVmax 5.7) in the descending colon, which was diagnosed as colon cancer by endoscopic biopsy (arrows)

**Fig. 4:**
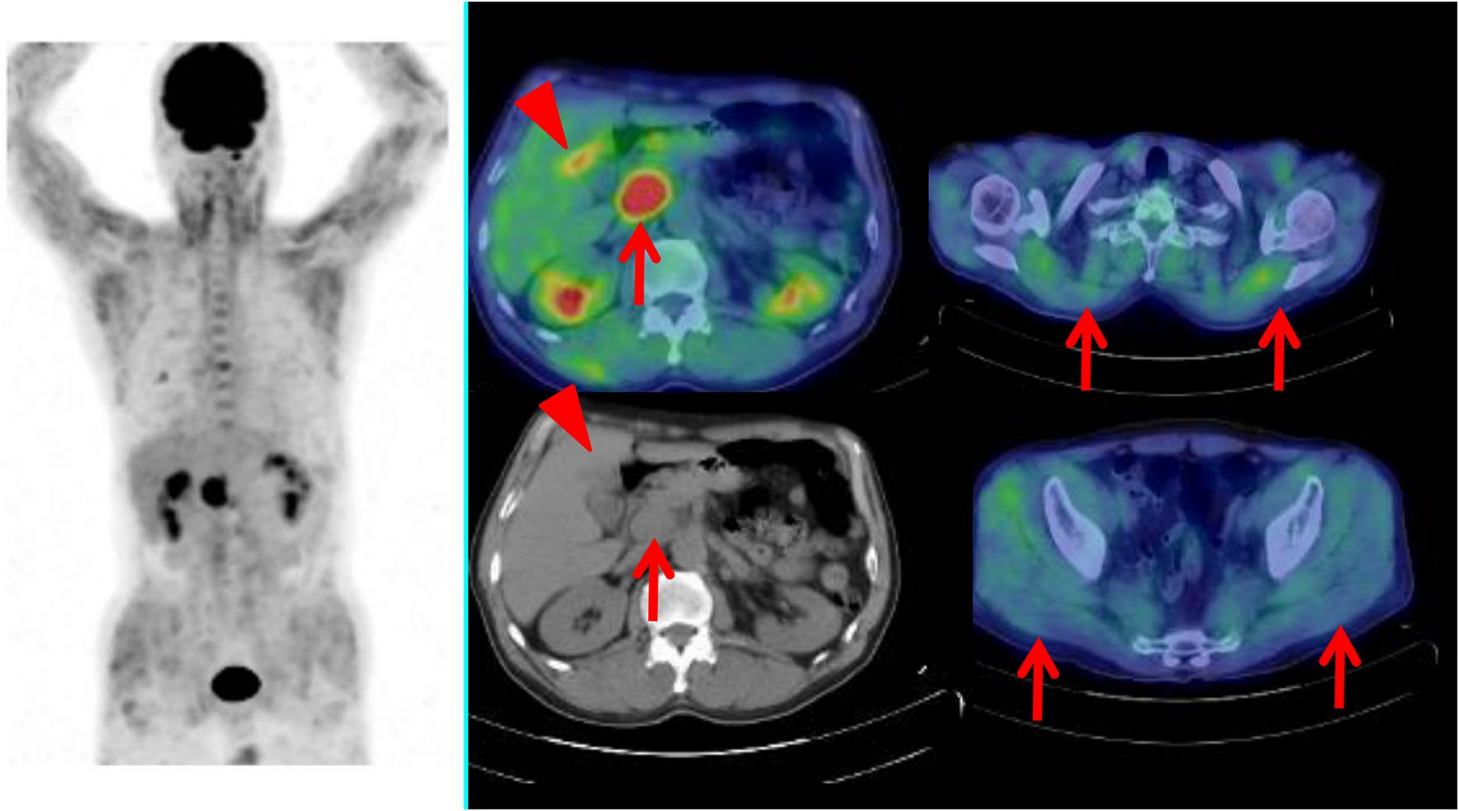
A dusky red rash developed systemically in the skin with itch in a 63-year-old male. Whole-body FDG PET maximum projection image showed heterogeneously increased FDG uptake in the systemic muscles. Cross-sectional FDG PET/CT images showed FDG-avid (SUVmax 3.1) gall-bladder cancer (arrow head) and metastatic lymph node near the root of the superior mesenteric artery (arrow), with heterogeneous but almost symmetrically increased FDG uptake in the muscle tissues (arrows)

**Fig. 5:**
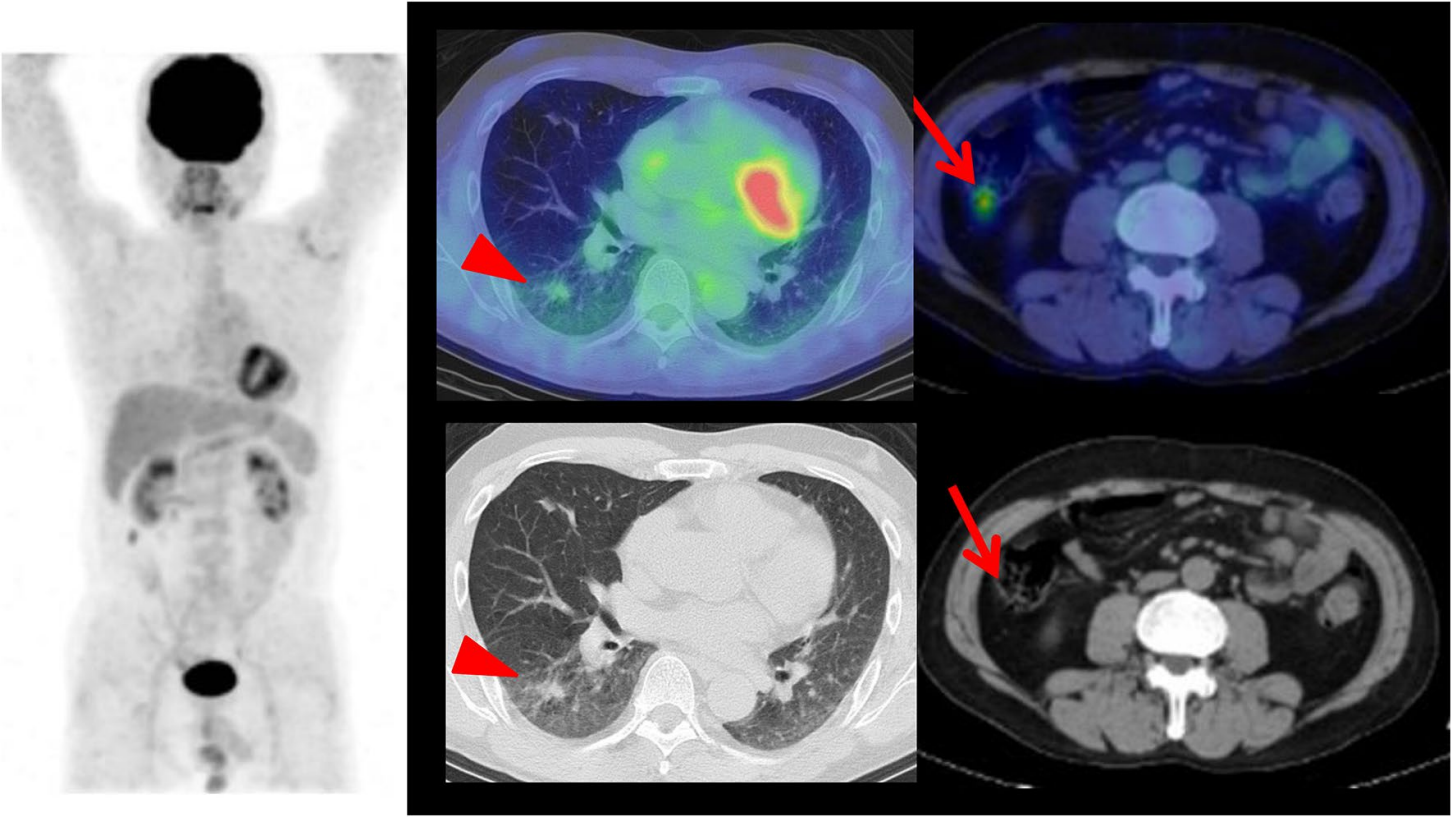
A 54-year-old male with dermatomyositis and interstitial pneumonia has been treated with corticosteroid. Whole-body FDG PET maximum projection image did not show increased FDG uptake in the systemic muscles probably due to the treatment effect. Cross-sectional FDG PET/CT images showed slightly increased FDG uptake (SUVmax 1.7) in the lung nodule of the right lower lobe (arrowhead), and focal FDG uptake (SUVmax 3.2) in the ascending colon which was diagnosed as colon cancer by endoscopic resection (arrow). Later, this patient had surgery of the right lung tumor of adenocarcinoma

Whole-body FDGPET/CT also has the excellent diagnostic performance for simultaneously detecting and diagnosing underlying or occult malignant neoplasms in DM patients [36–42]. It is comparable to a wide panel of extensive screening investigations in ability to detect cancers, and a single FDG PET/CT scan may potentially negate the need for numerous investigations [35, 41]. In our institute, among the patients with various PD, DM patients have the most frequently undergone whole-body FDG PET/CT to search internal malignancies. In our DM patients, 12(48%) out of 25 subjects examined showed FDG-avid malignant neoplasms, including 5 colon cancers (Fig. 3), 3 lung cancers, 2 breast cancers, 1 gastric cancer, 1 gallbladder cancer (Fig. 4), 1 thyroid cancer, 1 uterine body cancer, and 1 malignant lymphoma. It is noteworthy that 3 of these patients had duplicated malignant tumors (colon and breast cancers, colon and lung cancers, and colon and thyroid cancer, respectively) (Fig. 5). Except for the patients who had been treated for DM lesions, all the patients concomitantly showed FDG uptakes predominantly in the proximal muscles. One of the previous studies showed FDG-avid various malignant neoplasms in 17 (22%) patients among the total of 75 DM patients [39]. Another study showed FDG-avid various malignant neoplasms in 7 (12%) patients among the total of 55 DM patients [36, 41]. One of our cases showed a combination of DM, interstitial pneumonia and lung cancer (Fig. 5). While it is well-known that DM often coexists with interstitial pneumonia, the rate of occurrence of such a combination is only 0.47%, and only 10 cases in total have been reported in Japan in the past 25 years [38].

### Other skin diseases classified as potential PD

Some other cutaneous diseases/symptoms could be associated with underlying neoplasms, but with less associative strength than PD earlier described [43–49] (Table 2).

**Table 2:** Other skin diseases classified as potential paraneoplastic dermatoses

Potential paraneoplastic dermatoses	Associated neoplasms
Pityriasis rotunda	Hepatocellular, gastric and oesophageal carcinoma, prostate cancer, chronic lymphocytic leukemia and multiple myeloma
Palmoplantar keratoderma	Oral or esophageal carcinomas
Pyoderma gangrenosum	Myelodysplastic syndrome, myeloma, paraproteinemia (IgA) and leukemia
Acquired ichthyosis	Lymphoproliferative disorders
Scleromyxedema	Myeloma, lymphoma and leukemia
Papuloerythroderma	Malignant lymphoma
Lichen planus	Thymoma, A variety of malignancy
Herpes zoster	Lymphoid malignancy
Chronic itch	Lymphoma and leukemia
	Non-small cell lung carcinoma

**Fig. 6:**
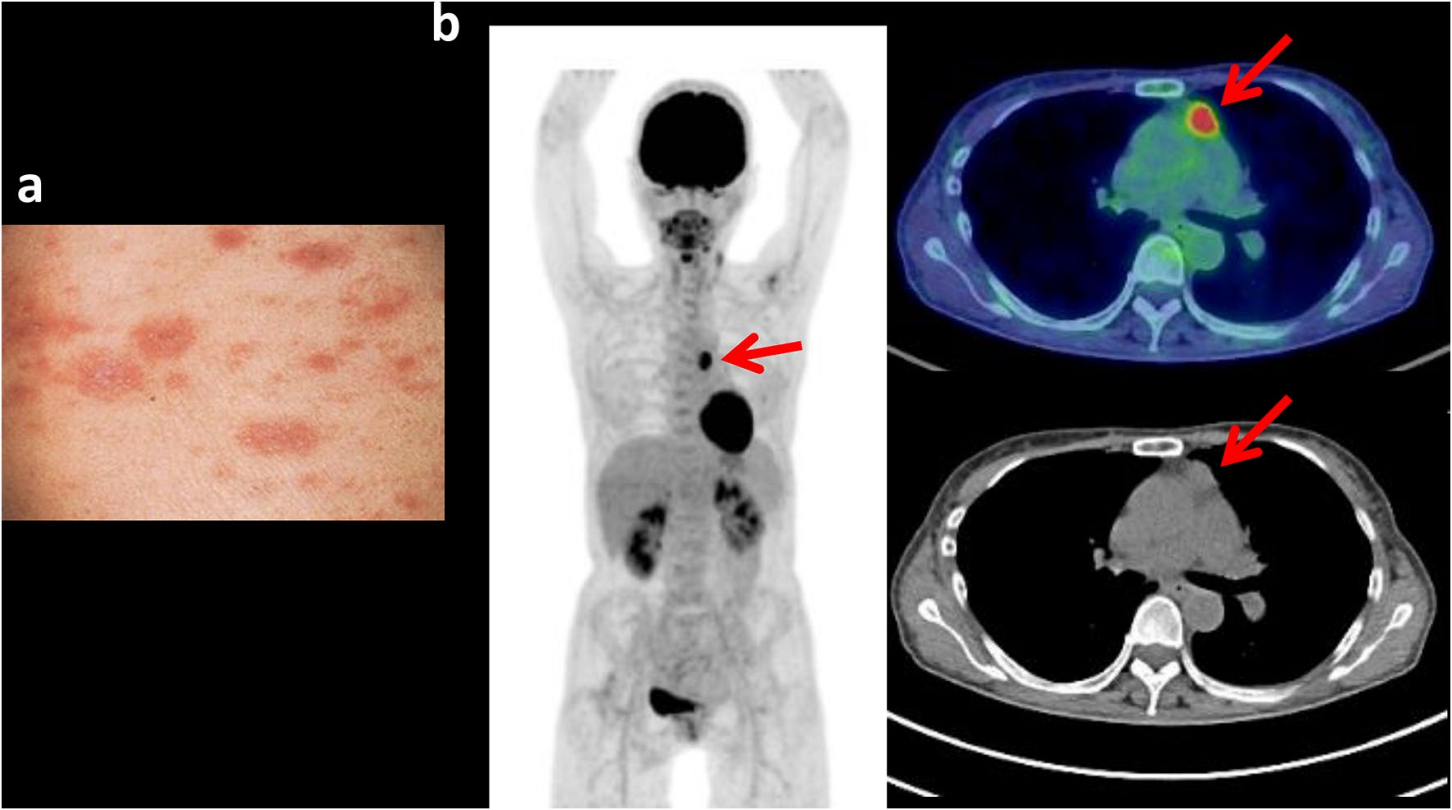
Close-up image of cutaneous lesion of lichen planus in the abdominal wall in a 75-year-old female (**a**). The cutaneous lesions began as erythematous patches on her hands and then spread to her feet, arms and legs, and trunk. Whole-body FDG PET maximum projection and cross-sectional images showed FDG-avid (SUVmax 7.4) mass in the right upper mediastinum (**b**; arrows), which was later diagnosed as thymic cancer by surgical resection. The cutaneous lichen planus lesions did not show any increased FDG uptake probably due to the thin and tiny lesions

Lichen planus is an immune-mediated disease which affects skin and mucous membranes and most frequently develops between the ages of 30 and 60 years. On the skin, it usually appears as purplish, itchy, flat bumps that develop over several weeks. In the mouth, vagina and other areas covered by a mucous membrane, it forms lacy white patches, sometimes with painful sores. It has been reported to be associated with a variety of disorders including different malignancies [46]. To our knowledge, there is no case report of FDG-avid neoplasms on FDG PET/CT in a patient with lichen planus. However, we have experienced a case of FDG-avid thymic carcinoma in a patient with lichen planus (Fig. 6).

**Fig. 7:**
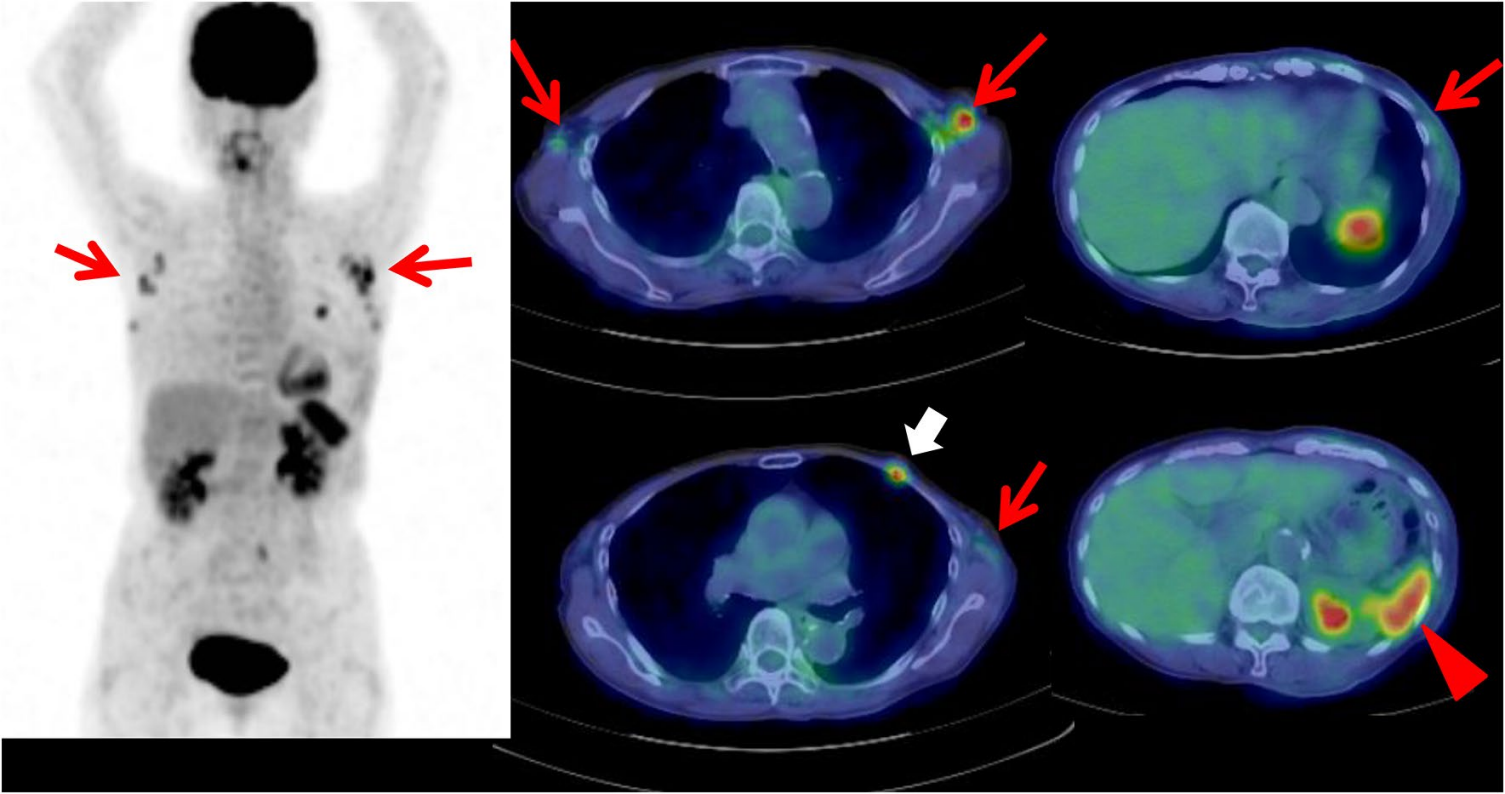
A 71-year-old female underwent FDG PET/CT scanning for left breast cancer, while she simultaneously suffered from herpes zoster lesions in left upper abdominal wall. Whole-body FDG PET maximum projection and cross-sectional images showed FDG uptake (SUVmax 1.9) in the active herpes zoster lesions in left abdomino-chest wall skin (arrows) and FDG uptake (SUVmax 4.8) in bilateral axillary lymph nodes (arrows), with FDG uptake (SUVmax4.7) in left breast cancer (white arrow). Diffusely increased FDG uptake (SUVmax5.6) was also seen in the spleen (arrowhead), which was probably associated with active herpes zoster. While FDG-avid lymph nodes of the left axillary region mimicked lymph node metastasis, the surgery of breast cancer after regression of active herpes zoster revealed the absence of metastasis in these lymph nodes

Herpes zoster may result from reactivation of latent virus in patients with immune system depression or dysfunction associated with underlying malignancy [47–50]. Increased risk of lymphoid malignancy in patients with herpes zoster also is reported [47]. Active herpes zoster lesions including lymphadenopathy show FDG uptake on FDG PET/CT, and may mimic lymph node metastasis in patients with malignancy [48–50]. We have experienced such a case in a patient with breast cancer (Fig. 7).

Although chronic itch (pruritus) is just symptom, it is the most common cutaneous symptom experienced by patients with various malignancies, such as hematological malignancies, bile duct/gallbladder carcinomas and liver carcinomas [45]. The presence of symptom of chronic itch occasionally has been documented in patients with these FDG-avid malignant lesions [51].

## Skin involvement of malignant neoplasms mimicking benign cutaneous diseases

The skin can be directly involved in malignancies, with the presence of tumor cells caused by direct tumor extension or metastasis, which may mimic some benign cutaneous diseases [4, 5]. Local skin involvement of malignant neoplasms has been reported in patients with gastric, breast, lung, and uterine cancers [4, 5]. Primary cutaneous lymphomas without involvement of lymph nodes, bone marrow or viscera are a heterogeneous group of lymphoproliferative neoplasms, and may mimic erythroderma or panniculitis [52–57]. Sixty five % of primary cutaneous lymphomas originate from mature T lymphocytes, 25% from mature B cells and the remaining part comprises neoplasms of natural killer cells [52–57]. They include mycosis fungoides, Sézary syndrome, primary cutaneous peripheral T cell lymphoma, primary cutaneous anaplastic large cell lymphoma, subcutaneous panniculitis-like T cell lymphoma, extra-nodal NK/T cell lymphoma, and nasal-type, etc. Mycosis fungoides is the commonest of a group of conditions known as cutaneous T cell lymphomas, and may progress very slowly. Most affected individuals initially develop skin lesions called patches, which are flat, scaly, pink or red areas on the skin that can be itchy, which may mimic psoriasis or other inflammatory cutaneous diseases. Affected individuals of mycosis fungoides have an increased risk of developing another lymphoma or other type of cancer.

**Fig. 8:**
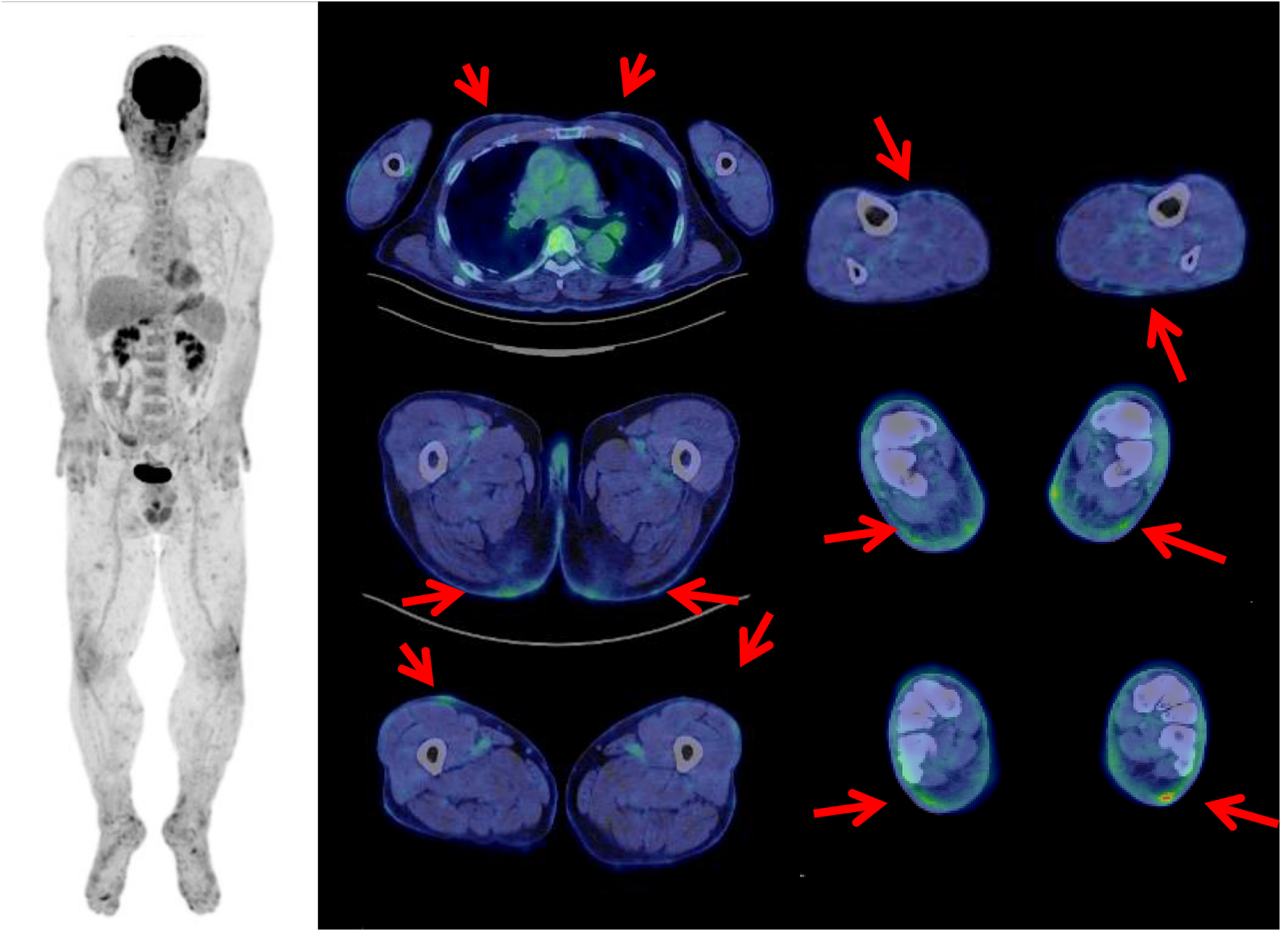
A 64-year-old male suffered rash-like skin redness, slightly raised or scaly round patches on the skin of the face, trunk and extremities. The biopsy of the skin lesion revealed the diagnosis of cutaneous peripheral T cell lymphoma. Whole-body FDG PET maximum projection and cross-sectional images showed FDG uptake (SUVmax 4.5) in the systemic skin lesions (arrows)

Only a few studies and case reports evaluate the potential role of FDG PET/CT in diagnosing primary cutaneous lymphomas [52–57]. Several studies indicate that FDG PET/CT is more accurate than CT in detecting both cutaneous and extra-cutaneous diseases of T cell lymphomas including mycosis fungoides [52–54]. We have experienced a total of 8 patients with primary cutaneous lymphomas (including 2 patients of mycosis fungoides), all of which showed FDG-avid cutaneous lesions (Fig. 8).

## Conclusion

PD can be clinical markers for underlying neoplasms. Recognition of PD and the following searching may lead to the diagnosis of internal malignant neoplasms including early stage ones. This article represented several cases of FDG-avid neoplasms in PD patients in our ten-year experiences, and reviewed the well-known and/or relatively common PD and their associated neoplasms, and the previously reported cases of FDG-avid associated neoplasms in these patients. FDG PET/CT can be expected as an excellent imaging modality for searching and detection of various underlying neoplasms in PD patients, while further accumulation and evaluation of the cases of FDG-avid-associated neoplasms in these patients are needed.
